# P03-009 - Experiences of in pediatric Behçet uveitis

**DOI:** 10.1186/1546-0096-11-S1-A204

**Published:** 2013-11-08

**Authors:** B Sozeri, S Yilmaz, S Mir, H Ates

**Affiliations:** 1 Pediatric Rheumatology, Ege University, Izmir, Turkey; 2 Ophtalmology, Ege University, Izmir, Turkey; 3 Peadiatric Nephrology, Ege University, Izmir, Turkey

## Introduction

Behcet disease (BD) is a chronic systemic inflammatory disease of unknown origin. Although,the clinical feature of juvenil BD is similar to adults, neurologic and gastrointestinal involvement have concluded more in juvenile BD.

## Objectives

To evaluate the efficacy and safety of immunosuppressive therapy including conventional therapy and anti-tumor necrosis factor-alpha (anti-TNF-α) agents in pediatric patients in Behcet uveitis.

## Methods

A retrospective study was made of 6 consecutive pediatric patients with BD. Inclusion criteria were fulfillment of the classification criteria of the International Study Group for Behçet Disease and onset of uveitis at 16 years of age or younger. The main outcome measures were sex, age at onset of uveitis, the initial symptom of Behcet disease, clinical ocular features, ocular complications and systemic treatment.

## Results

Four patients were female, 2 male were. Mean age at onset of uveitis was 11.8±3.2(7 to16) years. The most common extra-ocular clinical manifestations were recurrent oral ulcer in all patients and arthritis in 4 patients(50%) and pseudo folliculitis in 3 patients(66.7%). Pan uveitis was bilateral in83.3%, retinal vasculitis and retinitis were seen in83.3% and100% of the involved eyes, respectively.Cataract, maculopathy, glaucoma and optic atrophy were seen in 36.4%,18.1%,18.1 and0.9 %of the involved eyes, respectively.

Treatment modalities applied to treat either uveitis or its complications were classified as topical, and systemic. Corticosteroid drops (dexamethasone 0.1%, prednisolone 1%) with frequent instillation and cycloplegic drops (cyclopentholate 1%) 3 times daily were used in eyes with panuveitis. Systemic corticosteroid treatment was performed to suppress acute inflammatory episodes.

The mean duration of oral corticosteroid therapy for the treatment of acute inflammatory conditions was 3.4± 0.5 months (range, 3-4). All patients had used conventional immunosuppressive (IS) agents including azathioprine and cyclosporine, and 4 (66.7%) patients had additionally used anti-TNF treatment to control panuveitis attacks. Before starting to anti-TNF agents, screening for latent tuberculosis was performed using the local guideline.The majority of the patients (n:3) received only ADA subcutaneous injections once in every two weeks, while the one patient switched from IFX to ADA due to loss of clinical response. Ocular manifestations (panuveitis and retinal vasculitis) responded rapidly and reduction in the number and dose of standard immunosuppressive agents in patients with adalimumab. Overall, mean treatment period for anti-TNF agents was 9.5 ± 4.1 (range 6 to 14) months. Considering the 8 eyes of 4 patients with these anti-TNF agents, basal uveitis relapse rate of 4.0±0.8 decreased to 0.5±1.0 (p<0.05) during fallow-up. In 2 patients who completed the first year of anti-TNF treatment without any relapses, anti-TNF treatment could be stopped only in a single case using ADA, while anti-TNF treatment had to be continued in other. No adverse effect requiring cessation of anti-TNF agents was observed.

## Conclusion

In line with the previous data, our findings also suggest that anti TNF alpha agents may be tried in the treatment of pediatric Behçet uveitis resistant to other therapeutic approaches.

## Competing interests

None Declared

